# New Insight into the Fluorescence Quenching of Nitrogen-Containing Carbonaceous Quantum Dots—From Surface Chemistry to Biomedical Applications

**DOI:** 10.3390/ma14092454

**Published:** 2021-05-09

**Authors:** Marek Wiśniewski, Joanna Czarnecka, Paulina Bolibok, Michał Świdziński, Katarzyna Roszek

**Affiliations:** 1 Physicochemistry of Carbon Materials Research Group, Faculty of Chemistry, Nicolaus Copernicus University in Toruń, Gagarina 7, 87-100 Toruń, Poland; pbolibok@umk.pl; 2 Department of Biochemistry, Faculty of Biological and Veterinary Sciences, Nicolaus Copernicus University in Toruń, Lwowska 1, 87-100 Toruń, Poland; j_czar@umk.pl; 3 Department of Cellular and Molecular Biology, Faculty of Biological and Veterinary Sciences, Nicolaus Copernicus University in Toruń, Lwowska 1, 87-100 Toruń, Poland; mswidzin@umk.pl

**Keywords:** carbonaceous quantum dots, nitrogen-containing quantum dots, photoluminescence, quenching, intracellular uptake

## Abstract

Carbon-based quantum dots are widely suggested as fluorescent carriers of drugs, genes or other bioactive molecules. In this work, we thoroughly examine the easy-to-obtain, biocompatible, nitrogen-containing carbonaceous quantum dots (N-CQDs) with stable fluorescent properties that are resistant to wide-range pH changes. Moreover, we explain the mechanism of fluorescence quenching at extreme pH conditions. Our in vitro results indicate that N-CQDs penetrate the cell membrane; however, fluorescence intensity measured inside the cells was lower than expected from carbonaceous dots extracellular concentration decrease. We studied the mechanism of quenching and identified reduced form of β-nicotinamide adenine dinucleotide (NADH) as one of the intracellular quenchers. We proved it experimentally that the elucidated redox process triggers the efficient reduction of amide functionalities to non-fluorescent amines on carbonaceous dots surface. We determined the 5 nm–wide reactive redox zone around the N-CQD surface. The better understanding of fluorescence quenching will help to accurately quantify and dose the internalized carbonaceous quantum dots for biomedical applications.

## 1. Introduction

The development of novel functional nanosystems, connecting effective bio-imaging with therapeutic agent delivery (theranostic approach), has recently become the most promising direction in nanomedicine and is rapidly expanding. Fluorescent nanomaterials are among the most frequently employed biomaterials in theranostics. They allow for the optical real-time imaging of cells and tissues, facilitate the intraoperative image-guided surgery, and fulfill the role of efficient and easy-to-track drug carriers [1,2,3,4]. Connection of a therapeutic compound to the surface of fluorescent nanomaterial allows not only for drug tracking, but also for its targeted delivery and facilitated uptake into cells. The latter is pivotal for anticancer drugs or siRNA intercalating into DNA structure, as they have to be efficiently internalized [1,5]. Furthermore, the nanocarriers are expected not only to transport the chemotherapeutic agents directly into tumor cells, avoiding normal tissues and reducing systemic toxicity, but also to protect cytotoxic drugs from degradation, increase their half-life, and reduce renal clearance [6,7]. To date, the scientific attention has been mostly focused on known anticancer drugs immobilized on inorganic quantum dots (QDs), which are synthesized as heavy metal salts. Well-established QDs contain toxic elements, such as Cd, Pb, Hg, Se, and Te. They meet most of the expectations for drug-delivery nanosystems; moreover, the excitation-dependent emission of quantum dots allows to follow their distribution within the cells or even in the whole body. They are preferably tested in vitro in different cancer cell cultures [6,8,9]. The main drawbacks of these inorganic materials, however, are their high toxicity and non-biodegradability. Therefore, the use of amorphous, biocompatible, and biodegradable carbon quantum dots (CQDs) solves the above problems, in the great extent, and becomes the promising perspective in theranostic applications. Fluorescent carbonaceous nanoparticles are newcomers in the family of carbon-based nanomaterials that have emerged during the past several years and gained much interest as potential alternative to conventional, inorganic quantum dots [2,10].

In addition to the fascinating photoluminescent properties, CQDs are water dispersible, chemically stable, cell permeable, and fairly biocompatible or at least non-toxic to the cells and tissues. The benefits of low-cost simple synthesis and environmental friendliness are not to be underestimated [10,11]. Two distinct approaches to the synthesis of carbonaceous quantum dots have been widely described: the graphitized quantum dots are synthesized from reduced graphene oxide as precursor, and the amorphous ones are obtained via the hydrothermal treatment of carbon-rich molecular precursors. The carbon source for the latter method can be inter alia carbohydrates [12], gelatin [13], soy milk [14], bovine albumin [15], and polyacrylamide [16]. Nitrogen-rich precursors, such as aminoacids, peptides, or proteins, allow us to obtain specific CQDs. The undisputable advantages of nitrogen-containing carbonaceous quantum dots (N-CQDs) are their extremely low toxicity and outstanding biodegradability, and thus, they appear to be ideal tools for biomedical applications [17,18]. CQDs were also reported to be hardly attacked by the immune system and easily removed from the body—advantages that enable them to be harmlessly implemented for cell imaging, in vivo imaging, diagnostics, and as drug delivery systems [19,20].

There are numerous in vitro and in vivo studies focused on the cell or tissue imaging through carbon-based-quantum-dots-mediated labeling or facilitated tracking of CQD-linked drugs [21,22,23,24,25]. However, the bio-imaging of the carbonaceous nanocarrier-based therapeutics, together with their precise quantification in the living cells, is still the key challenge on their way from bench to bedside use. The underestimated obstacle can be for example the quenching phenomena of CQD fluorescence, that distorts the determination of the actual CQD concentration but is not often taken into consideration [24]. The quenching and recovery of the fluorescence of CQDs is mainly used for analytes detection [20]. These applications are underpinned with the principle that interactions between analytes and CQDs decrease the fluorescence by quenching phenomenon or increase fluorescence by suppressing the quenching effect. Quenching mechanisms include static quenching, dynamic quenching, energy transfer, photo-induced electron transfer (PET), and inner filter effect (IFE) [26,27]. The energy transfer is divided into Förster resonance energy transfer (FRET), Dexter energy transfer (DET), and surface energy transfer (SET). Static quenching occurs when a non-fluorescent complex is formed via the interaction between CQDs and quencher. Dynamic quenching can be explained as an effect of the excited state to the ground state return. The collision between the quencher and CQDs due to energy transfer or charge transfer occurs in this process [27]. Regardless of the mechanism, one has to realize that fluorescence quenching of CQDs is common in biological systems, thus making the quantitative analyses difficult.

In this work, we studied the physicochemical properties crucial for the biomedical use of N-CQDs. We aimed at elucidation of their concentration-dependent photoluminescence, stability, entry into different cells, and explanation of the N-CQD fluorescence quenching phenomenon occurring inside the cells. Our results indicate that N-CQD fluorescence intensity measured inside the cells was lower than expected based on carbonaceous dots extracellular concentration decrease. We elucidated the mechanism of quenching and identified the potential intracellular quenchers. Based on the fluorescence measured in cell lysates and culture medium, we indicated that a decrease in the N-CQDs fluorescence in the extracellular environment better reflects the quantity of material endocytosed. To the best of our knowledge, there are no reliable quantitative analyses of CQDs endocytosed/absorbed into the cells in the literature. This issue is extremely important in terms of different cell absorption ability on one hand, and accurate cell-targeted drug dosage on the other. The precise quantification of CQD-based therapeutics inside the cells is crucial for determination of efficient dose and increases the success rate of treatment.

## 2. Materials and Methods

### 2.1. Nitrogen-Containing Carbonaceous Quantum Dots Synthesis

**Figure 1:**
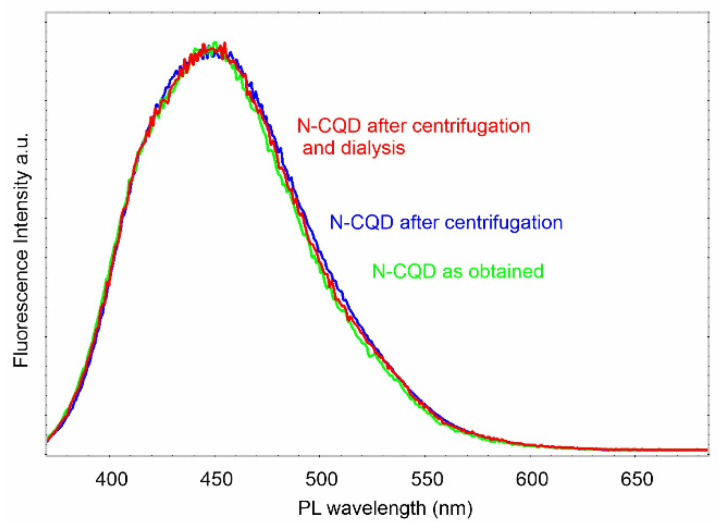
Fluorescence spectra of N-CQD samples subjected to different purification processes (excited at 360 nm).

Nitrogen-containing carbonaceous quantum dots (N-CQDs) were prepared via hydrothermal treatment of gelatin at 200 °C for 6 h, under 19 MPa. After cooling down to room temperature, the obtained brown solution (N-CQDs suspension) was ready for further examination. We compared the fluorescence spectra for the as-obtained sample and two others subjected to further purification processes (centrifugation at 15,000 *g* for 30 min and dialysis). Analysis of the spectra clearly indicates the lack of any impurities, as presented in Figure 1.

### 2.2. N-CQD Physicochemical Characteristics

Elemental analysis of prepared N-CQD samples was performed by energy-dispersive X-ray method (EDX). The equipment used for the EDX analysis was LEO Electron Microscopy Ltd., England, model 1430 VP. 

The High Resolution Transmission Electron Microscopy (HRTEM) images were taken by using a transmission electron microscope F20X-TWIN (FEI-Tecnai, Norcross, GA, USA) operated at 200 kV. The drop of sample solution was placed on a Cu-grid coated with an ultrathin amorphous carbon film and then dried under ambient conditions.

The morphology of N-CQD dispersed on Si wafers was analyzed at room temperature, in air, using a microscope with a scanning SPM probe of the NanoScope MultiMode type (Veeco Metrology, Inc., Santa Barbara, CA, USA) which operated in a tapping mode.

Fourier-transform infrared (FTIR) spectroscopy data were acquired by using a Vertex V70 (Bruker Optic), in ATR mode techniques (single reflection using diamond crystal), in the frequency range 6000–15 cm^−1^.

The fluorescence spectra were measured with a fluorescence spectrometer RF-5001PC (Shimadzu, Japan). Excitation maximum was experimentally established at 360 nm. Fluorescence decay curves and photoluminescence (PL) absolute quantum yield (QY) for as-obtained N-CQDs were measured at room temperature. UV–Vis spectra of N-CQDs were acquired by using a Jasco 660 spectrometer in the range of 200–800 nm.

### 2.3. Effect of pH, Concentration and NADH on the N-CQDs Fluorescence Quenching 

The prepared N-CQD aqueous solutions were adjusted to the various pH values by adding 2 M HCl or NaOH solution. The self-quenching of N-CQDs was tested in the concentration range of 1–5000 µg/mL. For NADH-mediated quenching experiments, to 1000 µg/mL of N-CQD solution, the aliquots of NADH were added up to final concentration 0.53 mmol/L. Then the corresponding emissive fluorescence spectra were measured upon excitation at 360 nm.

### 2.4. In Vitro Cell Culture

Human cervical cancer (HeLa) cell line was purchased from Sigma-Aldrich, Darmstadt, Germany. The human lung epithelial carcinoma cells (A549 cell line) were purchased from ATCC collection, whereas mouse mesenchymal stem cells (MSCs) were obtained from Life Technologies, Poland. Cells were grown at 37 °C, under humidified atmosphere with 5% of CO_2_. According to the manufacturer protocols, different culture media were used as follow: Ham’s F-12 medium containing 10% fetal bovine serum (FBS), 100 U/mL penicillin, and 100 μg/mL streptomycin (for A549 cells); DMEM-HG (high glucose) containing 10% fetal bovine serum (FBS), 100 U/mL penicillin, and 100 μg/mL streptomycin (for HeLa cells); and DMEM-LG (low glucose) containing 10% fetal bovine serum (FBS) and 50 μg/mL gentamycin (for MSCs). A volume of 25 μL containing 1 × 10^4^ cells was seeded to each well of a 24-well plate 24 h before the experiments were started.

### 2.5. Viability Assay and EC50 Determination

The N-CQD influence on the cell proliferation and viability was assayed, using the MTT (3-(4,5-dimethylthiazole-2-yl)-2,5-diphenyl tetrazolium bromide; Sigma Aldrich, Darmstadt, Germany) test. The appropriate concentrations of N-CQDs in culture medium were added to the growing cells in a 24-well culture plates and cultured for the selected time (usually 24 h). 

Then, 500 µL of 1 mg/mL MTT solution in a suitable culture medium was added to each well. After 1 h of incubation at 37 °C, the solution was removed, 500 μL of dimethyl sulfoxide (DMSO; Sigma Aldrich, Darmstadt, Germany) was added to each well, and the plates were shaken for 10 min. The absorbance was measured at the wavelength of 570 nm, with the subtraction of the 630 nm background, using a microplate reader. The viability of cells (relative to 100 % viable cells in control without N-CQD addition) was used to calculate the half-maximal effective concentration (EC50) of N-CQDs for different cell lines.

### 2.6. Microscopic Examination

The cells cultured on microscopic glasses, with or without N-CQDs, for 24 h were fixed in 4% paraformaldehyde (Polyscience) in phosphate-buffered saline (PBS), for 20 min at, room temperature. Next, the preparations were washed in PBS. The observations were carried out with an Olympus BX50 fluorescence microscope. An UPlanFI 100× (numerical aperture, 1.3) oil immersion lens and narrow-band filters U-MNU2 (excitation filter “BP360-370”, emission filter “BA420”, dichromatic mirror “DM400”) were used. The results were registered with an Olympus XC50 digital camera and Cell^B^ software (Olympus Soft Imaging Solutions GmbH, Münster, Germany).

### 2.7. Fluorescence Analyses in Cells and in the Extracellular Environment

Evaluation of the N-CQD amount endocytosed by cells was performed after 24 h of culture in the presence of 50, 100, 200, and 300 μg/mL N-CQDs. The cell monolayer was lysed through repeated freeze/thaw cycles. The fluorescence intensity in cell lysates and media collected from the culture plates was measured at an excitation wavelength of 360 nm and emission at 450 nm. The amount of internalized material was calculated based on the differences between N-CQD fluorescence in control samples (without cells) and post-culture media, and normalized to the number of cells. 

## 3. Results and Discussion

**Figure 2:**
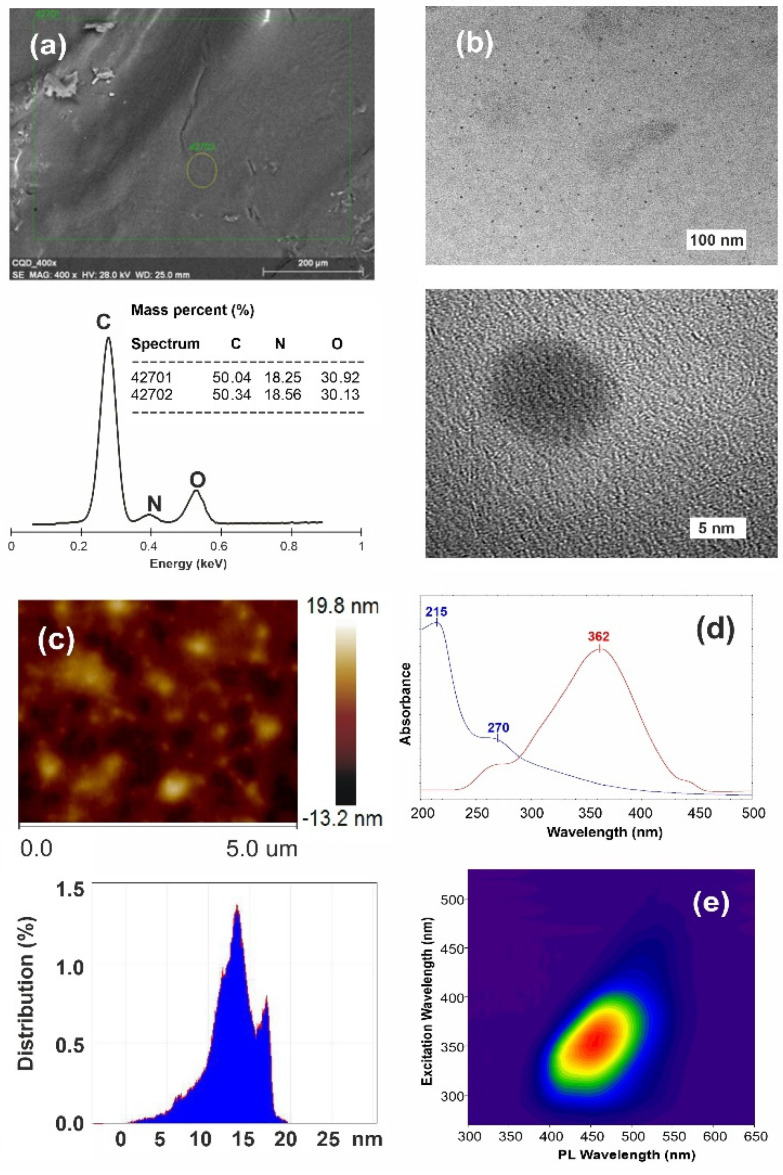
Characteristics of as-obtained N-CQDs: (**a**) elemental analysis based on EDX measurements, (**b**) HRTEM images, (**c**) AFM image with particle size distribution, (**d**) UV–Vis absorption spectrum (blue) and fluorescence excitation (red, λem = 450 nm), and (**e**) 2D fluorescence topographical map of the N-CQD.

The green, low-cost, and nitrogen-rich precursor, i.e., gelatin, was subjected to the one-pot hydrothermal process without adding any strong acids, oxidizers, or metals. The facile and environmentally friendly procedure resulted in the nitrogen-containing carbonaceous quantum dots (N-CQDs) containing 18.4% nitrogen (Figure 2a), and possessing almost uniform particle size below 20 nm, as presented in the Figure 2b,c. 

AFM analysis (Figure 2c) revealed a narrow size distribution, predominantly in the range of 10–15 nm. From this analysis, one can conclude that the gelatin-derived N-CQD materials are indeed the quantum-sized carbonaceous dots. The UV–Vis absorption spectrum of the N-CQDs (Figure 2d) displays two characteristic peaks at 215 and 270 nm attributed respectively to n-π* and π-π* transitions of C-X (X = N,O) and C=C bounds [28].

The quantum yield of the synthesized N-CQDs is about 17% relative to quinine sulfate (54% in 0.1 N H_2_SO_4_). The value is typical for N-containing carbon quantum dots [29,30,31]. The 2D fluorescence topographical map (Figure 2e) of the N-CQDs shows one, though indistinct, contour appearing with an emission maximum in the range of 430–470 nm, depending on the excitation wavelength. It depicts clearly that the excitons, generated in the fluorophore centers, get trapped and relaxed at distinct surface functionalities before recombination. Such red-shift of the emission peak depends on the distribution of different sizes N-CQDs and on the surface concentration of emission points [32].

**Figure 3:**
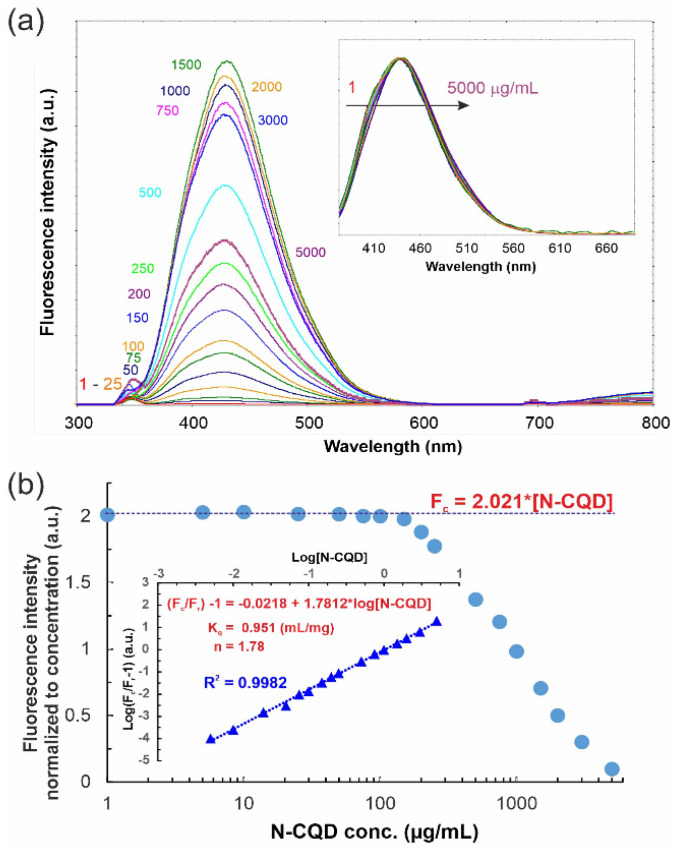
(**a**) Fluorescence spectra for different concentrations of synthesized N-CQDs (the numbers denote the concentration in µg/mL) indicate that, for the concentrations above 1500 µg/mL, the intensity decreases with increasing N-CQD concentration; inset shows normalized spectra showing lack of the emission band shifting. (**b**) Normalized to concentration fluorescence intensity as a concentration function; inset shows modified Stern–Volmer plots for quenching of various concentrations of N-CQD solution. Fc are the fluorescence intensities extrapolated in accordance to Lambert–Beer law while Fr means real observed intensities.

Similar to the majority of fluorescent substances, N-CQDs exhibit evident concentration-dependent self-quenching effects [30,33]. With the rise in concentration, the fluorescence intensity increases reaching a maximum for c.a. 1500 µg/mL N-CQDs, and then decreases (Figure 3a). As shown in Figure 3b, N-CQDs at an appropriately low concentration (below 150 µg/mL) exhibit perfect linear dependence of fluorescence intensity and concentration, revealed as a horizontal line in Figure 3b (with the value of 2.021 mL/µg for fluorescence intensity normalized to adequate concentration). In this concentration range, the Lambert–Beer law is matched perfectly. Further increase in concentration above 200 µg/mL shows deviation from this value (real fluorescence intensity (F_r_) decreases) as the self-quenching becomes meaningful. Therefore, we have defined a theoretical value for fluorescence intensity (F_c_) calculated as extrapolation of L–B law.

Based on the fact that (i) the fluorophore and quencher are attached to the same molecule, (ii) self-quenching rises with the concentration (Figure 3b), and (iii) overlapping between the emission and absorbance spectrum (Figure 2d) occurs, the Förster resonance energy transfer (FRET) mechanism is the most probable reason for observed fluorescence light screening. In other words, carbonaceous quantum dots are considered to consist of a dense carbon core and effective fluorophores dispersed at their surface. Once the fluorophores come too close to each other (e.g., due to increase in concentration), transient excited-state interactions lead to fluorescence auto-quenching or the formation of non-fluorescent ground-state species may be anticipated. This statement is confirmed by positive deviation from linearity in Stern–Volmer relation (data not shown) and the ideal linearity shown in inset in Figure 3b. The bimolecular rate constant of the fluorescence quenching process due to a short-range interaction of species (K_q_) becomes concentration dependent with the K_q_ value calculated as 0.951 L/g. Noteworthy, the obtained number of binding sites (n = 1.78) indicates that there is more than one binding center on the N-CQD surface.

**Figure 4:**
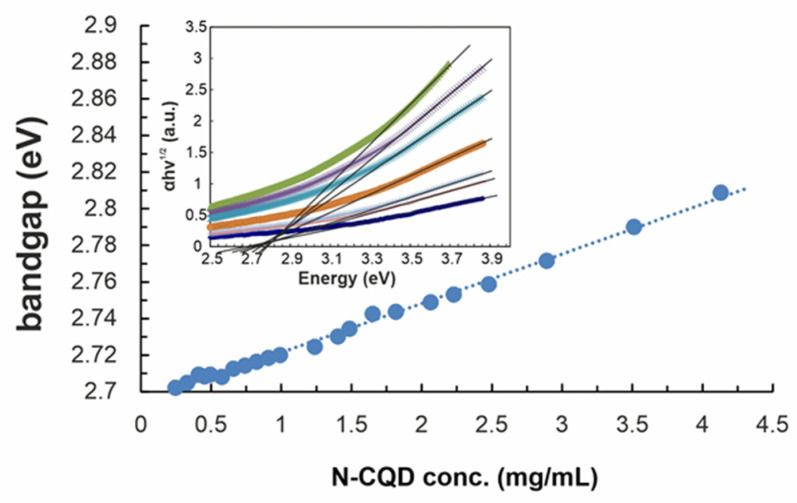
The correlation of band-gap and N-CQD concentration. Inset: the Tauc plot calculated from concentration-dependent UV–Vis spectra.

Localization of bulk electrons can be verified through the band gap (E_g_) analysis (see Figure 4). The values were determined from Tauc’s plot (inset in Figure 4) of (Ahν)^n^ for n =, i.e., for direct transitions, versus photon energy (hν), wherein A means absorbance from UV–Vis spectra. The E_g_ for the lowest N-CQD concentration (0.25 mg/mL) was calculated to be 2.70 eV and increases gradually when N-CQD concentration rises. As E_g_ increase is very well described with linear function, it is obvious that quenching process is the first-order reaction. The abovementioned relations confirm the semi-conductive properties of synthesized N-CQD and stay in good agreement with the literature reports. CQDs obtained from other precursors and with distinct methods but having similar nitrogen content reveal the same optical properties [34].

**Figure 5:**
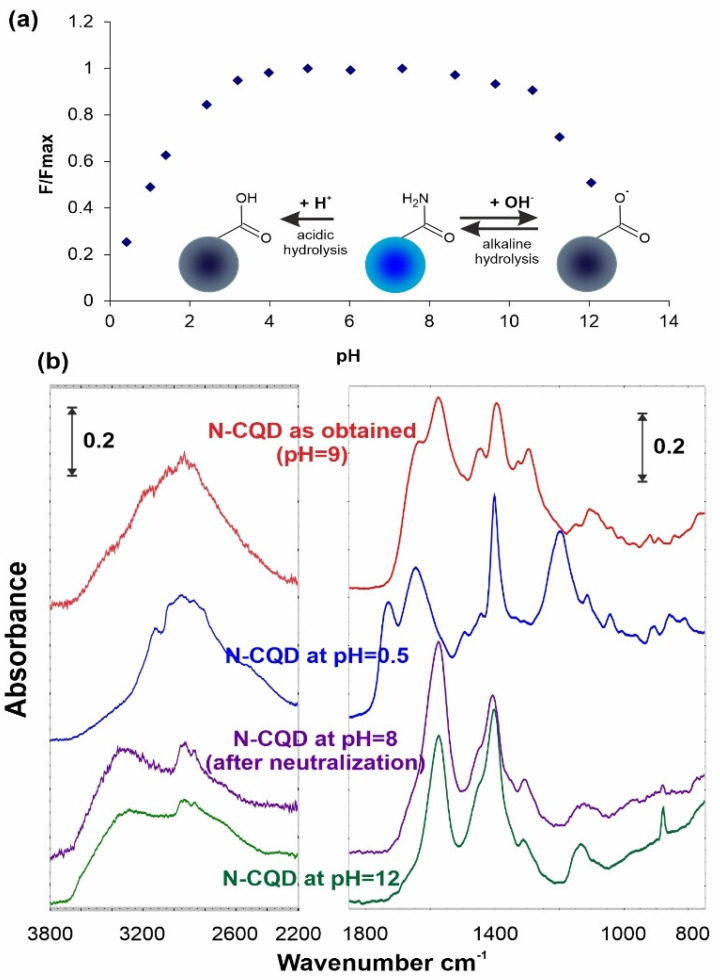
Effect of pH on (**a**) N-CQD fluorescence intensity and (**b**) N-CQD surface functionalities.

Another factor strongly influencing the CQD quenching is pH of the solution. Fluorescent probes employed in biological systems should remain stable at different pH values. The fluorescence intensity of tested N-CQDs (Figure 5a) shows wide-range pH stability from pH = 3 to pH = 10, while only significant changes of pH value resulted with decreased fluorescence intensity. In order to explain these phenomena, the functional groups of the N-CQDs were analyzed by the FTIR method in the ATR technique, and the infrared spectra of the samples were compared. Figure 5b presents the spectra of N-CQDs samples: as-obtained (note that pH of suspension equals 9), and exposed to acidic (pH = 0.5 from HCl solution) and alkaline environment (pH = 12 from NaOH solution).

During the hydrothermal treatment, mainly the dehydration and cyclization processes occurred which affected all observed functionalities. Spectral analysis (Figure 5b) reveals the presence of characteristic IR signals at 1770–1700 cm^−1^ for C=O stretch, at 1600–1500 cm^−1^ for N–H bending, and at 1500–1100 cm^−1^ for the C–O, C–C, and C–N stretch. It indicates that functional groups in source material are converted into amide groups (amide-I at 1635 cm^−1^, amide-II at 1573 cm^−1^, and amide-III in the range of 1480–1250 cm^−1^) by the hydrothermal process. The broad absorption bands in the range of 3700 to 2200 cm^−1^ are assigned to ν(O–H) and ν(N–H). All these groups are responsible for the hydrophilicity and colloidal stability of tested material in aqueous medium [35,36]. It should be noted that no absorption bands characteristic for aromatic compounds (stretching vibrations of C–H of aromatic rings over 3000 cm^−1^) were observed in the FTIR spectra of N-CQDs.

The observed spectral changes after immersing the N-CQD samples in strongly alkaline solution (pH = 12) causes disappearing of abovementioned IR signals and appearance of COO^−^ bands. The signals at 1572 and 1402 cm^−1^ clearly indicate that alkaline hydrolysis of surface amides has occurred. Apparently different spectrum was obtained after N-CQD soaking at pH = 0.5. The IR signals at 1730, 1401, and 1198 cm^−1^ can be attributed to surface –COOH functionalities. To prove this statement, the IR spectrum registered after neutralization perfectly covers with the one obtained at alkaline treatment. Additionally, the disappearance of 1645 cm^−1^ band proved that it origins from Zundel bend surface acidic structures [37]. Moreover, spectral changes in the –OH signals range and the appearance and disappearance of H-bonds fully confirm the above statements. It is obvious that, in both strongly acidic and basic environments, amide structures undergo the hydrolysis process, in which surface carboxyl groups are formed.

The above properties of N-CQD inspired us to study their applicability as fluorescent carriers of drugs, genes or other bioactive molecules. Three different model cell lines were chosen and compared in terms of the cytocompatibility and internalization of N-CQDs. Mesenchymal stem cells (MSC) are normal, healthy cells isolated from murine bone marrow, while lung adenocarcinoma A549 and cervical cancer HeLa cell lines are cancerous epithelial cells of human origin. Viability tests proved the expected cytocompatibility of N-CQD even at high concentrations; the viability of A549 cells and MSC was not reduced considerably after 24 h in vitro culture in the presence of maximal tested N-CQD concentrations (Table 1).

The half-maximal viability decrease, representative for material toxicity, was only achieved after 72 h of cell exposure to N-CQD concentrations exceeding 1 mg per mL. The half-maximal effective concentration (EC_50_) for HeLa cells, calculated after 24 h of exposure, exceeded 2000 µg/mL, and in comparison with other carbonaceous materials, it means that synthesized quantum dots can be safely used in a plethora of biomedical applications. 

**Figure 6:**
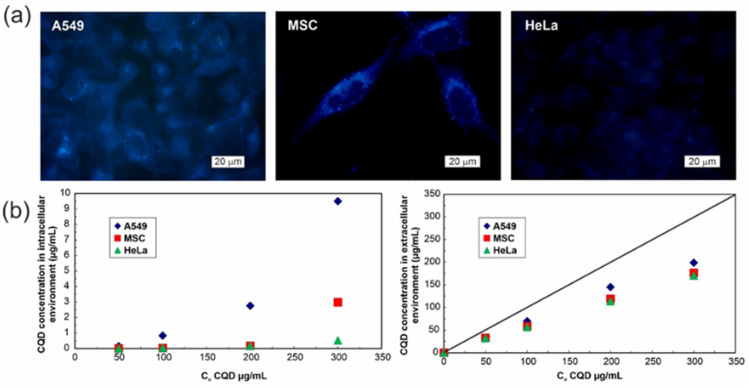
(**a**) Fluorescence microscopy images of A549, MSC, and HeLa cells after N-CQD absorption for 24 h in in vitro culture; (**b**) fluorescence-based determination of N-CQD concentration in cell lysates (left panel), and in the extracellular environment (right panel). Solid line corresponds to the initial N-CQD concentration in control samples (media without cells), and the difference between initial and measured N-CQD concentration indicates the amount of endocytosed material.

We aimed at shedding more light on the issue of N-CQD absorption by studied cell lines and quantitative determination of carbonaceous quantum dots internalized into the intracellular environment. The thorough analysis of microscopic images (sometimes used to assess the amount of fluorescent nanomaterial inside the cell (e.g., see Reference [24]) indicated some differences between cell lines that can be ascribed to their distinct absorption capabilities (see Figure 6a). From the micrographs one can conclude that HeLa cells have endocytosed less nanomaterial particles than two other cell lines (Figure 6a). It stays in accordance with the spectrofluorimetric analysis of cell lysates—the measured fluorescence corresponds with the concentration of 0.52, 2.98, and 9.50 µg/mL for HeLa, MSC, and A549 cells, respectively (Figure 6b left panel), and could have indicated relatively low rate of N-CQD endocytosis into the cells.

Surprisingly, N-CQD fluorescence intensity in the cells (from micrographs or lysates) is lower than the expected carbonaceous dots concentration calculated from the N-CQD decrease in extracellular milieu the fluorescence analysis of the extracellular environment (measured in culture media) specifies similar for all cell types, noticeable decline in N-CQD concentration by over 100 µg/mL after 24 h (see Figure 6b, right panel). In the applied concentration range, neither self-quenching nor pH-responsiveness give an elucidation to the underestimated quantity of endocytosed N-CQDs.

**Figure 7:**
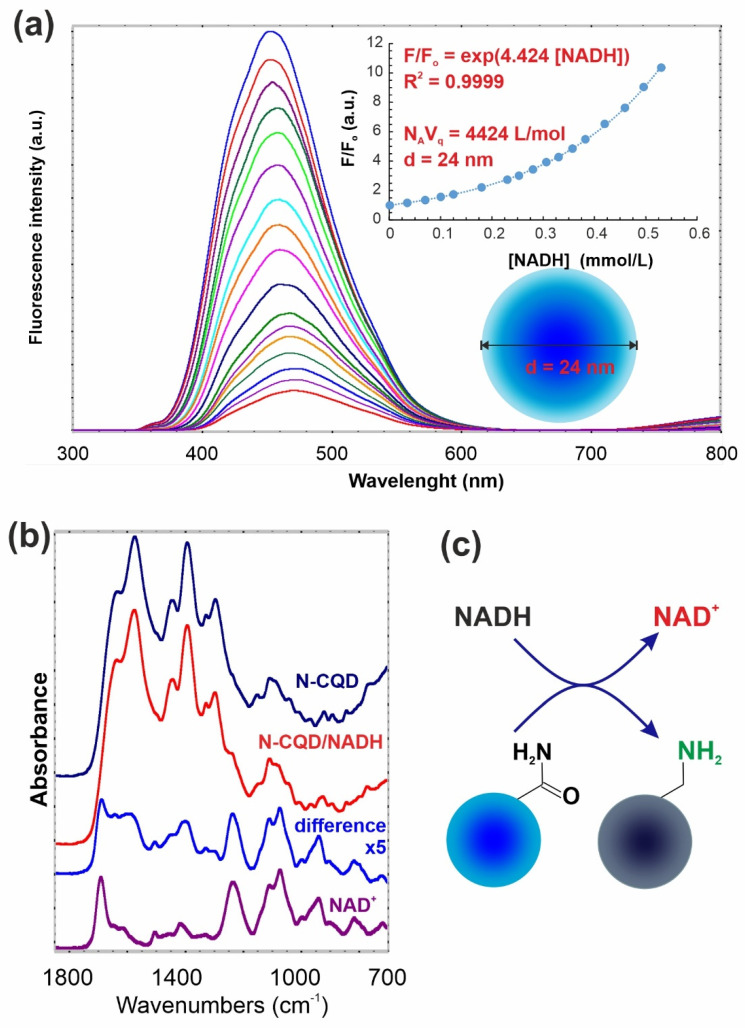
(**a**) Fluorescence emission spectra of N-CQD and their quenching by sequential addition of reduced form of nicotinamide adenine dinucleotide (NADH) aliquots. Inset shows Stern–Volmer plots for quenching with various concentrations of NADH. F_o_ is the fluorescence intensity of initial sample, while F means observed intensities; (**b**) FTIR spectra of NAD^+^ and N-CQD/NADH complex, and blue spectrum is the differential one; (**c**) schematic representation of the processes underlying the observed phenomenon.

To clarify this phenomenon, we have thoroughly examined different agents and conditions that influence the fluorescence quenching in the intracellular microenvironment, and we found that reducing agents, e.g., the reduced form of β-nicotinamide adenine dinucleotide (NADH), act as strong quenchers of N-CQD fluorescence—Figure 7a. During the process, the intense bands of surface –NH_2_ and C–C functionalities appeared respectively at 1600 and 1400 cm^−1^ (Figure 7b), indicating that surface primary amines are formed. 

Moreover, characteristic bands attributed to adsorbed NAD^+^ are also observed. Unfortunately, 1700 cm^−1^ signal from NAD^+^ masks the diminishing of –C=O signal. A collisional quenching process and the subsequent formation of non-fluorescent carbonaceous dots, can be explained in this case by the occurrence of concurrent, multiple quenching processes [38]. When energy transfer from a donor to nearby acceptors occurs via the electron exchange mechanism within the sphere, the Perrin model provides an approximate description [38], as shown in inset of Figure 7a. We can consider the redox process, where N-CQDs as nano-sized spheres are reduced, and NADH becomes oxidized (see Figure 7b,c). Thus, the calculated from Perrin’s equation radius of effective quenching is higher than the size calculated from AFM or HRTEM analysis (Figure 2b,c), indicating that reactive redox zone stretches approximately 5 nm beyond the N-CQD surface.

β-Nicotinamide adenine dinucleotide, NAD^+^, and its reduced form, NADH, are ubiquitous biomolecules found in all eukaryotic cells. Together with glutathione (GSH) and other reducing agents, they are essential for maintaining cellular redox homeostasis. The total concentration of NAD^+^ and NADH in the majority cells fits in the range of 10^−6^ M to 10^−3^ M and the ratio between them varies from 1 to 700 [39,40]. Typically, cytoplasmic and nuclear NAD^+^/NADH pools are more sensitive to changes in redox status than the mitochondrial NAD^+^/NADH reservoir, which is steadily maintained [41]. Intracellular NAD^+^ and NADH levels are also related to cell proliferation rate, oxidative stress, cell aging, and tumor development. 

The distinct concentrations of NADH and other reducing agents in different types of cells will strongly influence their capability to quench CQD fluorescence. Some authors [23], based on the fluorescence intensity in the cells, determined the uptake of CQDs into the intracellular environment. Unfortunately, there is no simple and universal rule that governs the relation between uptake and quenching. High metabolic or proliferation rate requires energy, primarily from reducing equivalents (NADH, FADH_2_) oxidation in mitochondria. Therefore, in fast proliferating cells NADH level might be low, and it is reflected by decreased quenching as in case of A549 cells. The more the intracellular environment is reducing, the lower the fluorescence observed inside the cells. However, it is worth noticing that cytosolic NAD^+^/NADH concentration can vary even in the same cells by the reason of fluctuations underpinned by the actual metabolic or redox status [41]. 

Carbon-based quantum dots (CQDs), by the reason of their biocompatibility and contrary to inorganic quantum dots, are widely suggested as the most beneficial fluorescent carriers of drugs, genes, or other bioactive molecules. The excitation-dependent fluorescence emission of carbon quantum dots allows to follow their distribution within the cells [3,21,22,23,24,25,31]. Connection of a therapeutic compound to the surface of the tailored nanomaterial should allow to decrease the systemic administration of drug and to minimize its side effects [6,7]. Thus, it is extremely important to know the exact amount of nanomaterials internalized into the cells, bearing also in mind the challenging process of their fluorescence quenching. To prevent the over-dosage of drug-loaded nanosystems, the thorough quenching studies have been required. Therefore, our study on the facile synthesis of amorphous N-CQDs with excellent biocompatibility and pH-resistant photoluminescent properties mainly addressed the factors influencing the fluorescence quenching of CQD and therefore distorting the precise quantification. 

## 4. Conclusions

Summing up, we have proven that nitrogen-containing carbonaceous quantum dots are semi-conductive materials with the capability of concentration-dependent self-quenching. On the other hand, their fluorescent properties are stable at a wide range of pH values, and only extreme pH conditions lead to hydrolysis of surface amide functionalities. These biocompatible quantum dots can be internalized by different cells; however, their fluorescence inside the cell is quenched. The insight into the mechanism of quenching suggests that NADH is one of the agents potentially responsible for this effect. Thus, the precise CQD quantification inside the cell requires the determination of concentration decrease from the extracellular environment. Since numerous research groups employ the carbon-based quantum dots for bio-imaging or drug delivery in a plethora of different cells, for in vitro and in vivo studies, the presented results are crucial for determination of CQD-based nanocarriers efficient dose and for increasing the success rate of treatment.

## Figures and Tables

**Table 1:** Half-maximal effective concentration of N-CQDs for tested cell lines after 24 and 72 h in culture.

Cell Line	EC_50_ Value (mg/mL) After 24 h	EC_50_ Value (mg/mL) After 72 h
A549	>5.0	2.98 ± 0.91
HeLa	2.08 ± 0.19	1.80 ± 0.29
MSC	>5.0	3.18 ± 0.89

## Data Availability

Not applicable.
